# Deubiquitinases: Novel Therapeutic Targets in Immune Surveillance?

**DOI:** 10.1155/2016/3481371

**Published:** 2016-08-14

**Authors:** Gloria Lopez-Castejon, Mariola J. Edelmann

**Affiliations:** ^1^Manchester Collaborative Centre for Inflammation Research, University of Manchester, 46 Grafton Street, Manchester M13 9NT, UK; ^2^Department of Microbiology and Cell Science, College of Agricultural and Life Sciences, University of Florida, 1355 Museum Drive, Gainesville, FL 32611-0700, USA

## Abstract

Inflammation is a protective response of the organism to tissue injury or infection. It occurs when the immune system recognizes Pathogen-Associated Molecular Patterns (PAMPs) or Damage-Associated Molecular Pattern (DAMPs) through the activation of Pattern Recognition Receptors. This initiates a variety of signalling events that conclude in the upregulation of proinflammatory molecules, which initiate an appropriate immune response. This response is tightly regulated since any aberrant activation of immune responses would have severe pathological consequences such as sepsis or chronic inflammatory and autoimmune diseases. Accumulative evidence shows that the ubiquitin system, and in particular ubiquitin-specific isopeptidases also known as deubiquitinases (DUBs), plays crucial roles in the control of these immune pathways. In this review we will give an up-to-date overview on the role of DUBs in the NF-*κ*B pathway and inflammasome activation, two intrinsically related events triggered by activation of the membrane TLRs as well as the cytosolic NOD and NLR receptors. Modulation of DUB activity by small molecules has been proposed as a way to control dysregulation or overactivation of these key players of the inflammatory response. We will also discuss the advances and challenges of a potential use of DUBs as therapeutic targets in inflammatory pathologies.

## 1. Introduction

Ubiquitination is a posttranslational modification (PTM) that involves the attachment of a ubiquitin molecule (~9 kDa) to a target protein. It is now well accepted that most of the cellular processes required for the maintenance of the cell homeostasis are regulated by the ubiquitin-proteasome system (UPS), including the regulation of innate immune signalling. Ubiquitination is mediated by a set of three enzymes, a ubiquitin-activating enzyme (E1), a ubiquitin-conjugating enzyme (E2), and a ubiquitin ligase (E3). Ubiquitin (Ub) is attached as a monomer or as polyubiquitin (poly-Ub) chains. This attachment occurs between a lysine group in the target protein and the carboxy-terminal glycine of Ub. The formation of Ub chains, however, occurs by formation of a bond between the carboxy-terminal glycine of Ub and one of the seven lysines (K6, K11, K27, K29, K33, K48, and K63) or the methionine (M1) present in the acceptor Ub molecule [1] allowing the generation of a wide variety of poly-Ub chains. Each poly-Ub chain type will influence the fate of the target protein differently. For instance, K48-conjugated Ub chains are considered a signal for protein degradation at the proteasome while K63 and M1 chains play important roles in signalling pathways [1]. Ubiquitination is a reversible process, and its reversibility is mediated by a family of proteases called deubiquitinases (deubiquitinating enzymes, DUBs). Keeping the balance between the addition and removal of ubiquitin moieties is crucial in maintaining cellular homeostasis and any disturbances in this balance can have adverse consequences for the cell.

## 2. Mechanisms of Regulation of DUBs

The human genome encodes ~100 DUBs that fall into five different families. There are four thiol protease families, the ubiquitin-specific proteases (USP), ubiquitin C-terminal hydrolases (UCH), ovarian tumour domain containing proteases (OTU), and Machado Joseph disease (MJD)/Josephin domain DUBs, and one zinc-metalloprotease group, the JAB1/MPN/Mov34 metalloenzyme family [2]. The main functions of DUBs are (i) generation/release of free ubiquitin from Ub precursors (*de novo* Ub synthesis), (ii) subtle editing of poly-Ub chains, and (iii) removal of the poly-Ub chains from substrates prior to degradation by proteasome-bound DUBs. DUBs, similarly to other proteases, are tightly regulated to avoid aberrant function that could be therefore detrimental to the cell. This is achieved by a combination of different layers of regulation at transcriptional and nontranscriptional levels.

As many other proteins DUBs are regulated at the transcriptional level. One of the best examples of transcriptional regulation is A20 (TNFAIP3), which is a member of the OTU family of DUBs. A20 expression levels are highly upregulated in a proinflammatory environment (i.e., in response to TLR4 activation) [3], reflecting its important role as a negative regulator in the inflammatory response, as we will discuss below. There are other DUBs, which are regulated in response to cytokines, including DUB1, DUB2, USP17 (DUB3), and OTUD-6B. DUB1 is specifically induced by IL-3, IL-5, and GM-CSF, while DUB2 is stimulated by IL-2. USP17 (DUB3) is involved in the regulation of cell growth and survival and it is regulated by the cytokines IL-4 and IL-6 [4]. Ovarian tumour domain containing 6B (OTUD-6B) is a DUB, whose expression in B lymphocytes is induced by secretion of IL-3, IL-4, IL-13, or GM-CSF. With prolonged stimulation, these cytokines have an opposite effect and instead lead to a decrease in OTUD-6B expression. A higher expression of OTUD-6B was associated with inhibition of cell growth, an increase in apoptosis, and arrest of cells in G1 phase [5].

DUBs are also heavily regulated at the activity level by different mechanisms. DUBs can acquire specificity due to recruitment factors that guide them towards a specific substrate. One example is USP10 that requires the protein MCPIP-1 (monocyte chemotactic protein induced protein 1) to interact with and deubiquitinate its substrate NEMO inhibiting the NF-*κ*B signalling cascade [6]. In other cases binding of the substrate actively contributes to DUB catalysis. For instance, USP7, whose catalytic triad exists in an inactive configuration, changes towards an active one upon ubiquitin binding suggesting that USP7 catalytic domain is only fully active when a ubiquitin molecule is correctly bound [7]. The presence of DUBs in molecular complexes is a common way to modulate their activity. This mechanism is essential for USP1, an inefficient enzyme alone, but its activity highly increases when bound to the WD40-repeat protein UAF-1 due to conformational changes that increase its catalytic activity [8]. USP1 is involved in DNA damage response, mainly in the Fanconi anemia (FA) pathway where it mediates the deubiquitination of FANCD2 and FANCI, a crucial step for the correct function of the FA pathway [9, 10].

Additionally DUB activity can be further adjusted by posttranslational modifications such as phosphorylation or ubiquitination [11]. For instance, phosphorylation of CYLD at Ser418 or USP7 at Ser18 led to an increase in the activities of these two DUBs. In the case of CYLD, this modification can be induced by LPS (lipopolysaccharide) or TNF-*α* (tumour necrosis factor) treatment and it can suppress its deubiquitinating activity on TNF receptor-associated factor 2 (TRAF2) [12]. Furthermore, this phosphorylation also occurs in dendritic cells (DCs) treated with LPS/Lex, which leads to a diminished activity of CYLD but not to its complete loss. This effect can be reversed by an inhibition of DC-SIGN signalling and also by depletion of IKK*ε* [13].

Changes in the cellular microenvironment can also have an effect on DUB activity. One example is the production of reactive oxygen species (ROS) generated during mitochondrial oxidative metabolism as well as in cellular responses to cytokines or bacterial invasion, which can inhibit cellular DUB activity by oxidation of the catalytic cysteine residue [14, 15].

To summarize, more than one regulatory mechanism can apply to certain DUBs, which highlights the importance of a fine and multifaceted control of DUB expression and activity.

## 3. Deubiquitination in TLR- and NLR-Mediated Immune Signalling

Innate immunity is triggered in response to either PAMPs, which are derived from microbial pathogens, or DAMPs such as ATP, cholesterol, or monosodium urate crystals. These danger signals are recognized by Pattern Recognition Receptors either at the cell membranes by Toll-Like Receptors (TLRs) or at the cytosol by receptors such as the NOD-like receptors (NLRs) [16]. Activation of these PRR receptors results in a variety of immune signalling cascades which lead to the induction of immune mediators and proinflammatory cytokines, such as TNF*α* or IL-1*β*, capable of triggering appropriate immune responses. These cytokines lead to the recruitment of immune cells to the site of infection or tissue damage, which initiates an inflammatory response. TLR- and NLR-mediated signalling is heavily controlled by the ubiquitin system, which plays an essential role in maintaining the appropriate regulation of these cellular pathways [1]. Although DUBs can be involved in many other inflammatory aspects, here we will discuss how DUBs contribute to TLR and NLR-induced pathways, focusing on the activation of two very important and related processes, NF-*κ*B pathway and inflammasome activation.

### 3.1. TLR Signalling

TLRs are transmembrane glycoproteins, which play a key role in the immune response against microbes. Ten human TLRs have been identified to date and they either localize to the cell surface (TLRs 1, 2, 4, 5, 6, and 10) or have endosomal localization (TLRs 3, 7, 8, and 9 [17]).

**Figure 1:**
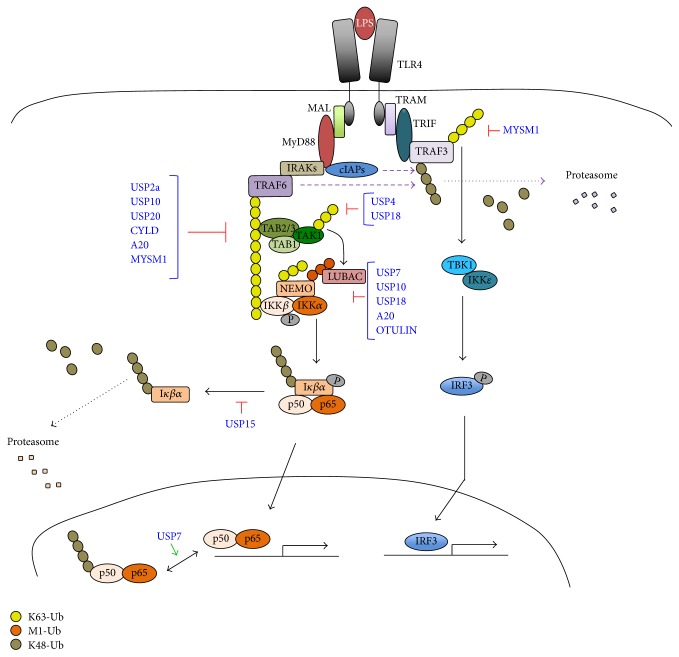
Regulation of TLR4 signalling by the ubiquitin-proteasome system. In MyD88-dependent signalling, TRAF6 and cIAP1/2s mediate K48 polyubiquitination and consequent degradation of TRAF3 by the proteasome. TRAF6 synthesizes K63 poly-Ub chains, which act as a scaffold for TAK1 and IKK complexes, TAB2/3 and NEMO. This occurs with the help of LUBAC, which leads to the linear ubiquitination of NEMO required for the recruitment of the IKK complex (IKK*α* and IKK*β*). As a result, TAK1 phosphorylates IKK*β*, which in turn phosphorylates I*κ*B and subsequently undergoes ubiquitination and proteasomal degradation. This event frees NF-*κ*B (p50/p65) to translocate to the nucleus and initiate transcription. Several DUBs (in blue) remove ubiquitin chains from TRAF6, NEMO, or NF-*κ*B, negatively regulating this signalling pathway. USP7 can also prevent NF-*κ*B degradation hence positively regulating transcription. MyD88-independent signalling occurs through TRAM/TRIF. In this case K63 poly-Ub chains are added to TRAF3, which consequently recruits the TBK1/IKK*ε* kinase complex. This phosphorylates IRF3 allowing nuclear translocation and initiation of transcription. The DUB MYSM1 can deubiquitinate TRAF3, controlling the extent of this signalling.

There are two distinguishable pathways of TLR signalling, one via the MyD88 (myeloid differentiation primary-response protein 88) and the second one via TRIF (TIR domain containing adaptor protein inducing interferon *α*/*β*) and apart from TLR3, most other TLRs are associated with the MyD88 pathway [18]. Ubiquitination is critically involved in optimal TLR-triggered MyD88 and TRIF signalling (Figure 1). TLR3 engagement induces the recruitment of TRIF and modification of TRAF3 with K63 poly-Ub, which consequently recruits the TBK1 (TRAF family member-associated NF-*κ*B activator-binding kinase)/IKK*ε* kinase complex. Finally, this cascade of events causes IRF3 activation and INF*γ* production. In contrast, TLR4 or TLR2 activation leads to the assembly of the MyD88 signalling complex, recruiting TRAF6, cIAP1, and cIAP2. These ubiquitin ligases mediate K48-linked poly-Ub of TRAF3, and TRAF3 is consequently degraded by the proteasome [19]. TRAF6 ubiquitin ligase activity is essential for the synthesis of K63-linked poly-Ub chains, which act as a scaffold to recruit other proteins required for signalling. TRAF6 K63-linked poly-Ub chains recruit both the TAK1 and IKK complexes through their respective ubiquitin-binding subunits, TAB2/3 and NEMO. This occurs with the help of the LUBAC ubiquitin ligase complex, which leads to the linear ubiquitination of NEMO required for the recruitment of the IKK complex (IKK*α* and IKK*β*). As a result, TAK1 phosphorylates IKK*β*, which in turn phosphorylates I*κ*B and subsequently undergoes ubiquitination and proteasomal degradation [20]. This allows NF-*κ*B to translocate to the nucleus from cytosol and regulate the transcription of a variety of target genes (Figure 1).

Deubiquitination also plays a key role in TLR signalling pathways by reversing the effect of ubiquitination and controlling the intensity of the immune response (Figure 1). Several DUBs have been identified to participate in the TLR signalling, the most studied and best characterized being A20 (TNFAIP3) and CYLD. A20 plays an essential role in restricting TLR signalling and maintaining immune homeostasis. A20 contains an OTU domain, which has DUB activity specific towards several NF-*κ*B signalling factors, such as TRAF6, RIPK1, or NEMO, which consequently leads to suppressed NF-*κ*B activation [21]. A20 is an unusual DUB because it encodes seven zinc-finger (ZnF) motifs, which confer E3 ubiquitin ligase activity on A20. This allows A20 to perform an editing function: in addition to removing K63-linked polyubiquitin chains from substrates such as RIPK1, A20 can introduce K48-polyubiquitin chains in the same substrate tagging it for a proteasomal degradation [21]. In addition to this, A20 can also regulate NF-*κ*B independently of its enzymatic activity. A20 can bind polyubiquitin chains through its ZnF domain allowing the interaction of ubiquitinated NEMO with A20. This ubiquitin-induced recruitment of A20 to NEMO is sufficient to block IKK phosphorylation by its upstream kinase TAK1 preventing NF-*κ*B activation [22]. In contrast CYLD is a tumour suppressor, whose loss leads to familial cylindromatosis, a skin tumour hereditary disorder, but that also controls NF-*κ*B activation. CYLD achieves this by specifically cleaving K63-linked poly-Ub chains and linear poly-Ub chains from RIPK1, TRAF2, and NEMO and similarly to A20 negatively regulates NF-*κ*B signalling [23].

USP7 was first identified as a herpesvirus associated protein, hence its alternative name HAUSP (herpesvirus associated USP). USP7 presents dual roles in the regulation of NF-*κ*B. It can regulate NF-*κ*B transcriptional activity in the nucleus, by deubiquitinating NF-*κ*B and preventing its degradation, hence increasing its transcriptional activity [24]. But USP7 can also act as a negative cytosolic regulator by deubiquitinating NEMO and consequently decreasing proteasomal degradation of I*κ*B*α*. This in turn retains NF-*κ*B in the cytoplasm and further suppresses NF-*κ*B activity [25]. These two reported and opposing roles suggest that USP7 can perform different functions roles, depending on substrate recognition or cellular localization, highlighting the tight activity control of this protease.

As previously mentioned, USP10 is required for mediated inhibition of NF-*κ*B activation. By mediating USP10-dependent deubiquitination of NEMO, MCPIP1 serves in a negative feedback mechanism for attenuation of NF-*κ*B activation [6]. TRAF family member-associated NF-*κ*B activator (TANK) interacts with both MCPIP1 and USP10, which leads to decrease in TRAF6 ubiquitination and the termination of the NF-*κ*B activation in response to TLR activation [26]. In accordance with this, depletion of USP10 is associated with TLR-triggered increase in NF-*κ*B activation [26].

USP18 is responsible for counteracting ISG15 conjugation and it is an important negative regulator of the IFN responses, thereby playing important roles in viral responses [27]. However, we now know that USP18 also mediates and regulates TLR-induced NF-*κ*B activation by cleavage of K63-polyubiquitin chains, but not K48 chains, of TAK1 and NEMO [28].

In addition to the DUBs described here there are several others implicated in the downregulation of the NF-*κ*B pathway upon TLR activation, although these are not well characterized. These include the USP family members USP2a, USP4, USP15, USP21, and USP31 and the member of the JAMM family MYSM1 and their substrates have been summarized in Table 1.

### 3.2. NLR Signalling

The NLR family presents a characteristic tripartite domain architecture with a variable C-terminus, a middle NACHT domain, and a Leucine Rich Repeat (LRR) N-terminus. The C-terminal LRR domain is involved in the ligand binding or activator sensing while the N-terminal domain performs effector functions by interacting with other proteins. NLRs are classified into four subfamilies according to their N-terminal domains: the acidic transactivation domain (NLRA), the baculoviral inhibitory repeat-like domain (NLRB) that includes NOD1 and NOD2, the caspase activation and recruitment domain (CARD; NLRC), and the pyrin domain (NLRP). NLRs can recognize a wide variety of ligands including pathogens, endogenous molecules, or environmental factors [29]. Their functions can vary and they are divided into four steps: inflammasome formation, signalling transduction, transcription activation, and autophagy [29]. Similarly to TLRs, NLR activation is also tightly regulated and PTMs play an important role here. Although ubiquitination in NLR signalling is well accepted, the role of DUBs in these pathways is just emerging.

#### 3.2.1. NOD1 and NOD2

**Figure 2:**
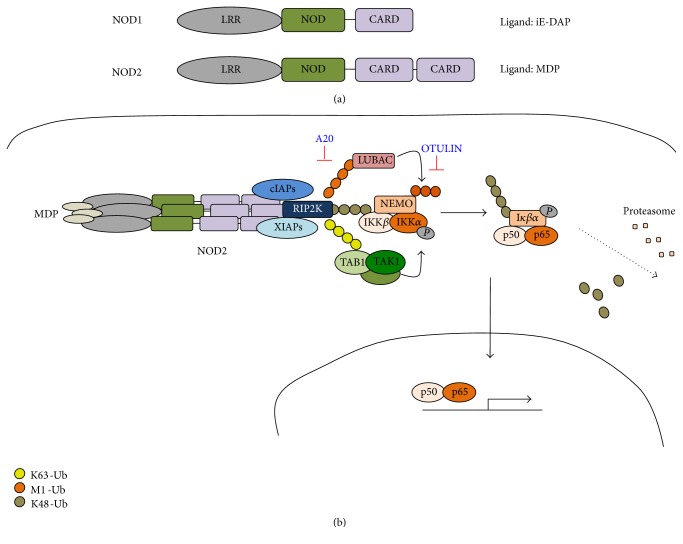
Regulation of NOD signalling by the ubiquitin-proteasome system. (a) NOD1 receptors recognize iE-DAP while NOD2 main ligand is muramyl dipeptide (MDP). (b) Similarly to NOD1, NOD2 receptors oligomerize upon ligand binding. This triggers the recruitment of RIPK2 to this complex and cIAP- and XIAP-mediated K63-ubiquitination of RIPK2. This allows the recruitment of TAK1/TAB2/TAB3 complex and LUBAC, which can also mediate the linear ubiquitination of RIPK2. TAK1 then phosphorylates IKK*β*, which in turn phosphorylates I*κ*B and subsequently undergoes ubiquitination and proteasomal degradation. This frees NF-*κ*B (p50/p65) to translocate to the nucleus and initiate transcription. Deubiquitinases A20 and OTULIN are negative regulators of these events by deubiquitinating K63 and M1 poly-Ub chains, respectively.

NOD1 and NOD2 receptors are important bacterial sensors, which recognize peptidoglycan (PGN). NOD1 senses the iE-DAP dipeptide, which is found in PGN of all Gram-negative and certain Gram-positive bacteria, while NOD2 recognizes MDP (muramyl dipeptide), the minimal bioactive peptidoglycan motif common to all bacteria (Figure 2(a)). Upon encountering with these ligands, NOD1 and NOD2 form oligomeric complexes, leading to the activation of NF-*κ*B and MAPK. IAPs (cIAP1, cIAP2, and XIAP) are central regulators of NOD1 and NOD2 signalling. Upon oligomerization RIPK2 is recruited to this complex. cIAP1, cIAP2, and XIAP contribute to K63-linked ubiquitination of RIPK2. This allows the recruitment of TAK1/TAB2/TAB3 complex and LUBAC, which can also mediate the linear ubiquitination of RIPK2, and further contributes to the NF-*κ*B and MAPK pathway activation by ubiquitination of NEMO [30, 31]. Ubiquitin can directly bind to the CARD domain of NOD1 or NOD2 and compete with RIPK2 for its association with these receptors, suggesting that ubiquitin might play a negative regulatory role [32, 33] (Figure 2(b)). A20 also plays a regulatory role in NOD2 signalling by deubiquitinating RIPK2 to control the extent of the inflammatory signals. A20-deficient cells present an amplified response to MDP, including increased RIPK2 ubiquitination and NF-*κ*B signalling [34].

One of the DUBs, which is relatively poorly characterized but which has been shown to play key functions in NOD2 signalling, is OTULIN. This protein specifically deconjugates linear (M1) poly-Ub chains assembled by LUBAC and in this way it modulates linear ubiquitination of LUBAC's substrates and provides fine-tuning of the initial activation of NF-*κ*B. By deubiquitinating RIPK2, OTULIN prevents NEMO binding and hence decreases its downstream signalling. Because LUBAC continuously ubiquitinates itself and other substrates, OTULIN plays an important role to avoid accumulation of Met1-Ub chains and overactivation of this pathway [35] (Table 1, Figure 2(b)).

#### 3.2.2. The Inflammasome

Another crucial function of NLR receptors is their contribution to the inflammasome. The inflammasome is a molecular complex, which consists of a sensor molecule (NLR, e.g., NLRP1, NLRP3, NLRC4, or NLRP6), an adaptor protein (ASC, apoptosis-associated speck-like protein containing a CARD domain), and an effector molecule (caspase-1) [36]. The main function of the effector molecule is to induce the cleavage and activation of the proinflammatory cytokines, IL-1*β* and IL-18. These proinflammatory proteins are synthesized as precursor molecules and require caspase-1 activation within the inflammasome in order to be released and cleaved and perform their biological activity. Activation of inflammasomes occurs in two steps. First, an NF-*κ*B mediated initial step leads to increased expression of NLRP3 and pro-IL-1*β*. Then an activating signal triggers rapid activation of caspase-1. Caspase-1 activation can be achieved by several K^+^-releasing molecules, including nigericin, crystals, or extracellular ATP through the activation of the ATP-gated P2X7 receptor (P2X7R) [37]. After the inflammasome is fully activated, it can lead to pyroptotic cell death, which can be distinguished from other cell death types by pore formation in the plasma membrane followed by osmotic cell lysis and finally the release of IL-1*β* and IL-18 [36].

**Figure 3:**
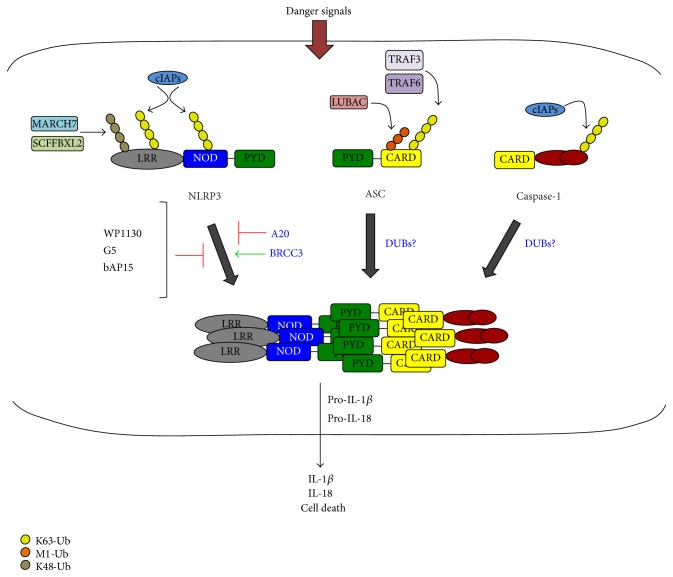
Regulation of the NLRP3 inflammasome activation by the ubiquitin-proteasome system. Assembly of the NLRP3 inflammasome complex occurs in response to a wide variety of danger signals including ATP, bacterial toxins, or particulate matter such as monosodium urate crystals. The ubiquitin ligases MARCH7 and SCFBXL2 add K48-linked poly-Ub chains to NLRP3 as a mean to control its levels by proteasomal degradation. cIAPs on the contrary add K63 poly-Ub chains to NLRP3 and caspase-1, contributing to the assembly of the complex. A20 also acts as a negative regulator of this complex. However, BRCC3 can deubiquitinate NLRP3, allowing it to form the complex and acting as a positive regulator of this pathway. TRAF3, TRAF6, and LUBAC also ubiquitinate ASC by K63 or M1 poly-Ub chains and this contributes to complex assembly. How other DUBs contribute to the assembly of the NLRP3 complex still remains unknown.

Given the important role of ubiquitin in signalling cascades derived from TLR and NLR activation, it is not surprising to find that assembly and activation of an inflammasome is also regulated by the ubiquitin system. Ubiquitination can regulate canonical inflammasome activation by modulation of three major components: NLR, ASC, and caspase-1. Ubiquitin ligases can also directly influence NLRP3 inflammasome activation. This can be exemplified by MARCH7, which promotes ubiquitination of NLRP3, and this causes its degradation upon dopamine stimulation as a mean to control inflammasome activation [38]. Another example is SCFFBXL2, whose activity is impaired upon LPS priming preventing NLRP3 ubiquitination and its consequent degradation [39] (Figure 3). Other ubiquitin ligases have also been involved in control of NLRP3 ubiquitination. For instance, TRIM30 can negatively regulate NLRP3 inflammasome by modulating the levels of ROS species in the cell. TRIM30^−/−^ macrophages produce higher levels of ROS and potentiate NLRP3 inflammasome activation; however the mechanisms by which TRIM30 controls this remain unknown [40]. However, TRIM33 is essential for cytosolic RNA-induced NLRP3 inflammasome activation. TRIM33 ubiquitinates DHX33, a cytosolic dsDNA sensor for NLRP3, allowing DHX33-NLRP3 interactions and consequent inflammasome activation [41]. Similarly to the NOD2 receptor activation, cIAP E3s are also involved in the inflammasome activation. Attenuation of cIAP activities, either by their deletion or by inhibition, triggers NLRP3 and caspase-1 activation as well as RIP3 kinase-dependent IL-1*β* processing and secretion [42].

On the other hand, cIAP1 and cIAP2 can attach K63-linked poly-Ub chains to caspase-1, thereby facilitating caspase-1 activation and IL-1*β* release [43]. Caspase-1 ubiquitination also occurs in response to the NLRP1 activator anthrax lethal toxin [43, 44] although the type of ubiquitin chains and whether this is a requirement for caspase-1 activation still remain unclear.

In addition to NLR and caspase-1, ubiquitin-mediated inflammasome activation can be also promoted by modification of the adaptor protein ASC. Activation of the inflammasome can induce autophagy as a mean to control inflammasome activation. In this situation, K63 poly-Ub modification of ASC allows for its interaction with the autophagic adaptor p62 and delivery of ASC to the autophagosome [45]. TRAF3 ubiquitin ligase ubiquitinates ASC, and abolishment of the target lysine (K174) prevents inflammasome activation and IL-1*β* release in response to viral infection [46]. Also, TRAF6-mediated ASC ubiquitination has been recently reported in response to far-infrared and proposed to constitute a mechanism, which dampens inflammasome activation in repair processes [47]. Interestingly ASC has been identified as a substrate of HOIL-1L, a member of linear ubiquitination complex LUBAC, and HOIL deficient macrophages present an impaired inflammasome response [48]. In line with this, macrophages deficient in SHARPIN, which is a different member of the LUBAC complex, are not able to mount an optimal inflammasome response [49].

All this evidence reveals that ubiquitination is an essential modification for the control of the inflammasome activation. It is then logical to assume that DUBs are important players of these regulatory mechanisms. This was first suggested by Juliana et al., who showed that NLRP3 is ubiquitinated in resting macrophages and that, upon cell activation with priming (LPS) and activating signals (ATP, nigericin, and MSU crystals), these ubiquitin chains are removed by DUBs, allowing activation of the complex [50]. This report was quickly followed by two other studies supporting these results [51, 52], and it was Py et al. who identified BRCC3 as the first DUB to be directly involved in inflammasome activation. These reports showed that inhibition of DUB activity with the DUB inhibitors bAP-15, WP1130, PR-619, and G5 blocks NLRP3 but not NLRC4 or AIM2 mediated IL-1*β* release and pyroptosis (Figure 3; Table 1). Moreover, a recent report has demonstrated that histone deacetylase 6 (HDAC6) negatively regulates NLRP3 inflammasome activation. HDAC6 interacts with NLRP3's ubiquitin-binding domain and treatment with the DUB inhibitor PR-619 results in an increased interaction of NLRP3 with HDAC6. The authors suggest this is due to an increased ubiquitination of NLRP3 and the consequent inhibition of NLRP3-dependent caspase-1 activation [53]. The ability of these DUB inhibitors to block inflammasome activation could explain the inhibitory effect of the compound Bay 11-7082 on NLRP3 inflammasome independently of its NF-*κ*B inhibitory activity [54] since this compound can inhibit components of the ubiquitin system, including DUBs [55, 56]. The other DUB, which has been directly implicated in the inflammasome activation, is A20. In contrast to BRCC3, A20 acts as a negative regulator of NLRP3 and suppresses inflammasome activation by restricting ubiquitination of IL-1*β* and NLRP3 activation [57, 58].

Given the fine-tuning and the layers of regulation required for both the inflammasome and DUB activation, it is quite likely to think that different DUBs might perform opposing functions pertaining to the inflammasome activation. Whether DUBs regulate the ubiquitination state of ASC or caspase-1 involved in the inflammasome assembly still remains unknown.

## 4. Pathogen Manipulation of DUBs to Control PRR Signalling

During pathogenesis, deubiquitinating enzymes are regulated both by microorganisms and by a host cell. Pathogens can exploit the host ubiquitin system by expressing their own ubiquitin-specific enzymes, and the host cell can up- or downregulate expression and/or activity of host DUBs [59].

First, an example of a pathogen-encoded deubiquitinase disturbing the host innate immune pathways is* Salmonella*'s AvrA, which is a DUB that facilitates inhibition of the NF-*κ*B pathway. AvrA leads to stabilization of I*κ*B*α* and prevents nuclear translocation of NF-*κ*B p65. Also, depletion of AvrA in* Salmonella* leads to significantly increased secretion of cytokine IL-6 in the host cell, which is dependent on NF-*κ*B pathway [60–63]. As a second example,* Chlamydia trachomatis* encodes two DUBs, ChlaDub1 and ChlaDub2, which are specific for ubiquitin but they also harbour deneddylating activity [64]. ChlaDub1 binds and stabilizes I*κ*B*α*, most likely via its deubiquitination, and finally this can lead to an inhibition of NF-*κ*B activation [65]. Since several known bacterial DUBs directly target important functions in the host immune system, development of selective inhibitors for pathogenic DUBs could be exploited as a therapeutic approach in the treatment of infections.

Bacterial infection can induce inflammasome activation in the host cell [36] and deubiquitination has been implicated in this process.* Salmonella* Typhimurium infection leads to changes in the activity of several host DUBs, such as USP4, USP5, UCHL3, and UCHL5, and increased activity of UCHL5 was found to contribute to the inflammasome activation during this infection [66]. Additionally, enteropathogenic* Escherichia coli* protein NleA associates with and interrupts deubiquitination of NLRP3, thereby repressing inflammasome activation [67].

## 5. Deubiquitinases and Inflammatory Disease

Accumulating evidence indicates that somatic mutations in DUBs are correlated with human disease. DUBs are genetically altered in many human cancers (i.e., CYLD, A20, or USP6) or contribute to the stability of oncogenes or tumour suppressors (i.e., USP7, USP8, or BRCC3) [68]. Here we will highlight DUBs with potential implications in immune disease although the scope for other DUBs contributing to disease is very high. Although many of the studies mentioned in this review have been performed* in vitro* in cell culture models, the involvement of DUBs in inflammatory responses has been also studied by using animal models, highlighting the relevance of these proteases in a relevant tissue and immune context (Table 1).

Mutations in the CYLD gene lead to a subtype of the benign cancer predisposition syndrome of skin appendages also known as Brooke-Spiegler syndrome, although inactivation or downregulation of CYLD is also observed in a variety of other cancers, including melanoma, and breast, colon, lung, breast, cervical, and, recently, prostate cancer. As previously mentioned CYLD can bind to NEMO and NF-*κ*B that have been identified as its substrates. It is possible that the negative regulation of NF-*κ*B mediated by CYLD contributes to its tumour suppression function given the increasingly recognized role for NF-*κ*B in cancer advancement. CYLD deactivation could provide specific advantage to tumour cells by enhanced NF-*κ*B signalling [69–71]. CYLD-deficient mice present abnormalities in their immune system. They show increased basal and induced NF-*κ*B activation and can develop autoimmune symptoms and colonic inflammation with features of human inflammatory bowel disease [72], and their inflammatory responses in response to pathogenic infection are potentiated [73].

A20 is an important negative regulator of immune response as we have mentioned before. Multiple mutations in the A20 gene have been identified; however no inheritable syndrome has so far been linked with A20 abnormalities. This could be explained if these mutations were developmentally critical. A20 mutations are strongly linked to autoimmunity, lymphomas, and asthma [74, 75], highlighting important differences to CYLD despite both targeting NF-*κ*B. This might be explained by different chain preference, K48 and K11 for A20 compared to the K63 and M1 chain preference showed by CYLD [68]. A20^−/−^ mice fail to regulate NF-*κ*B responses, develop severe inflammation and are hypersensitive to LPS or TNF*α* leading to premature death [76]. Cell specific ablation of A20 has revealed important knowledge about the contribution of A20 to disease pathogenesis and generated very useful mouse models for several conditions like rheumatoid arthritis, lupus erythematosus, or inflammatory bowel disease [75].

USP18 has been thoroughly studied in the context of viral responses, since it regulates protein ISGylation in response to viral infection. However Liu et al. also demonstrated that USP18 deficient mice are resistant to experimental autoimmune encephalomyelitis (EAE) [77]. This study proposes that USP18 regulates TAK1-TAB interaction and is hence necessary for Th17 differentiation and autoimmune response.

DUBs can contribute to disease not only by mutations, but also by an altered expression or activity. An example of this is USP7, whose increased activity mediates the deubiquitination and destabilization of a number of critical tumour suppressors, including p53 or PTEN, and is by inference an oncogenic prosurvival protein. The interrelationship between p53, USP7, and MDM2 ubiquitin ligase is quite unique and complex. USP7 can deubiquitinate and stabilize p53, but interestingly it can also deubiquitinate and stabilize MDM2 indirectly leading to p53 destabilization and its degradation by the proteasome [78]. USP7 also interacts and stabilizes the ICP0 ubiquitin E3 ligase of herpes simplex virus (HSV), which is required for the effective initiation of the lytic cycle, facilitating lytic viral growth [79]. USP7 can also interact with other viral proteins, such as the EBNA1 protein of the Epstein-Barr virus (EBV) [80] and the Viral Interferon Regulatory Factor 1 (vIRF1) of a Kaposi sarcoma herpesvirus protein [81]. In addition, and as mentioned before, USP7 plays a role by regulating NF-*κ*B signalling [24, 25]. Unfortunately USP7^−/−^ mice are embryonically lethal explaining the lack of* in vivo* studies to further characterize the role of USP7 in immune responses and associated pathologies [82].

## 6. Modulating DUB Activity as a Novel Inflammatory Therapeutic Approach

Given the importance of DUBs in inflammatory and other pathological responses, it is certainly easy to think of DUBs as potential therapeutic targets, whose modulation could be beneficial for inflammatory conditions. However, up to date there are no DUB targeting compounds that have been approved for clinical use, either in the inflammatory or in cancer context. The identification and success of inhibitors that target other elements of the ubiquitin system suggest that altering inflammation by targeting the ubiquitin system, including DUBs, could be a viable approach to develop novel anti-inflammatory treatments. An example of successful development of UPS inhibitors has been achieved with the proteasomal inhibitors Bortezomib or Carfilzomib, which have been effected in multiple myeloma treatment [83]. Another compound, MLN4924 (Nedd8-E1 enzyme inhibitor), has reached phase I clinical trials [84] and SMAC mimetics, which promote proteasomal degradation of cIAPs, have recently proved to work in cancer patients through phase I clinical trials [85].

DUB targeting drugs present a great potential as novel therapeutic agents. DUBs present the advantage of being druggable targets since they have a catalytic domain, and unlike other UPS members, such as the E3 ubiquitin ligase family with approx. 600 members, targeting the DUB family seems an achievable target. Given the clear evidence of the contribution of DUBs to disease there is a considerable effort put into the development of compounds that modulate DUB activity. Intensive research is being channelled to develop selective DUB inhibitors, which could be applied to such diseases like cancer, neurological and inflammatory disorders, or infectious disease.

Despite these intensive efforts and great advances in the DUB field, selective compounds have not reached clinical trials yet. Although no DUB-selective compound has yet reached clinical trials, the field is moving fast and in the right direction. MISSION Therapeutics is developing new DUB inhibitors that present good oral bioavailability and low EC_50_s in cell viability assays. Proteostasis Therapeutics in collaboration with Biogen is developing very promising USP14 inhibitor series, while Genentech and Almac might be developing a new therapeutic generation of USP7 inhibitors [86, 87].

This is due to two main challenges: first not all DUBs work in the same manner hence different strategies need to be followed to develop these compounds and second we do not completely understand how these enzymes function and/or are regulated. In addition, many of the studies, which address DUB functions, have been developed in* in vitro* systems using either isolated proteins or cell lines that are not relevant to function or disease. This might not reflect the reality of DUB behaviour in a tissue-specific context and more work has to be developed using* in vivo* mice models and primary human cells. To achieve this, new and more powerful tools are required, including in-cell based assays to discriminate selective DUB function and cytotoxicity and the development of inducible mouse models, which would allow for the study of tissue-specific DUB functions. It is fundamental that basic research and drug development teams work in close collaboration to allow the success of these compounds [86, 87].

Based on our actual knowledge on DUBs thinking that not all DUBs will be good therapeutic targets is likely, since some of them might share more than one substrate, which play opposing roles in different tissues or be essential to maintain homeostasis and health. For instance, targeting USP7 in the oncology context would be a good therapeutic strategy [88]; however we need to very carefully consider the possible effects of inhibiting USP7 on the inflammatory response to the tumour. Whether this would be detrimental or beneficial still remains unknown. Similarly, we could argue that potentiating A20 function in an inflammatory context would be a plausible treatment; however more detailed studies in the consequences of this approach are required. The presence of DUBs in pathogens causative of disease, such as virus, bacteria, or parasites, has also highlighted the possibility of developing DUB inhibitors, which specifically target the pathogen and not the host. In the following years new knowledge emerging from ongoing research will allow scientists to discern those that constitute good targets and offer promising new alternatives to existing therapeutics.

## 7. Concluding Remarks

Immune responses are strongly regulated by the addition and removal of ubiquitin molecules, and although the roles of E3 ubiquitin ligases in these signalling pathways are well established, it is still unclear how DUBs contribute to PRR signalling. The advances in this field due to novel tools and approaches including advanced mass spectroscopic techniques, ubiquitin linkage-specific antibodies, and structural and biochemical studies will provide new insights into the regulatory mechanism of immune signalling molecules by DUBs and vice versa.

Since the involvement of DUBs in several inflammatory conditions is clear, development of potent and selective DUB-specific inhibitors or agonists could provide new therapeutics to treat these conditions. For instance, given the high regulation of NOD1/2 by ubiquitin and the contribution of NOD mutations to inflammatory diseases such as inflammatory bowel disease (IBD) or Crohn's disease, it is possible that DUBs could be used as a target in NOD-associated inflammatory conditions.

Similarly to the kinase research area 20 years ago the DUB field is in its infancy. There are many challenges that remain to be solved to further advance our understanding of DUB function, specificity, and activity and to develop compounds that inhibit this activity. However, the field is advancing quickly, and hopefully new highly selective DUB inhibitors will be developed very soon.

## Figures and Tables

**Table 1:** DUBs involved in TLR, NOD1/2, or inflammasome activation. Knock-out mouse available for these DUBs has been indicated. Mouse model validation of target in which these mice have been used to demonstrate their function on that substrate. This table does not include studies where these mice have been used in other models of inflammation.

DUB	PRR	Target	KO mouse available	Mouse model validation of target	Ref.
USP2a	TLR	TRAF6	Yes	No	[89]
USP4	TLR	TAK1	Yes	No	[90]
USP7	TLR	NF-*κ*B, NEMO	No, lethal	No	[24, 25, 82]
USP10	TLR	NEMO, TRAF6	No, lethal	No	[6, 26, 91]
USP15	TLR	I*κ*B*α*	Yes	No	[92]
USP18	TLR	TAK1, NEMO	Yes	Yes	[28, 77]
USP20	TLR	TRAF6	No	No	[93]
USP21	TLR	RIPK1	Yes	No	[94, 95]
USP25	TLR	TRAF3	Yes	Yes	[96, 97]
USP31	TLR		Yes	No	[98]
A20	TLR	TRAF6, RIPK1, NEMO	Yes	Yes	[3, 76, 99, 100]
	NOD1/2	RIPK2		Yes	[34]
	NLRP3 inflammasome			Yes	[57, 58]
Cezanne	TLR	TRAF6	Yes	Yes	[101, 102]
OTULIN	TLR	NEMO	No, lethal	No	[103, 104]
	NOD2	RIPK2			[103, 105]
CYLD	TLR	RIPK1, TRAF2, NEMO	Yes	Yes	[12, 72]
MYSM1	TLR	TRAF3, TRAF6	Yes	Yes	[105, 106]
BRCC3	NLRP3 inflammasome	NLRP3	No	No	[51]
